# Optimum Sowing Date and Genotype Testing for Upland Rice Production in Brazil

**DOI:** 10.1038/s41598-018-26628-6

**Published:** 2018-05-29

**Authors:** Samuel Ferrari, Paulo Pagliari, Juliana Trettel

**Affiliations:** 1 Departamento de Fitotecnia, Unesp, Universidade Estadual Paulista, Campus de Dracena, Rodovia Comandante João Ribeiro de Barros, km 651, Bairro das Antas, CEP, Dracena, SP 17900-000 Brazil; 2 Department of Soil, Water, and Climate, University of Minnesota, Southwest Research and Outreach Center, 23669 130th, St. Lamberton, MN 56152 USA; 3 Departamento de Fitotecnia, Unesp, Universidade Estadual Paulista, Campus de Registro, Avenida Nelson Brihi Badur, 430, Vila Tupy, CEP, Registro, SP 11.900-000 Brazil

## Abstract

A better understanding of widespread agricultural practices adopted in the region of Ribeira Valley, São Paulo, Brazil for upland rice (*Oryza sativa* L.) production is needed. The objectives of this study were to determine the optimum sowing date and highest yielding genotypes for rice production. Treatments included four upland rice genotypes (ANA 5011, AN-Cambará, Moti-Amarelo, and Moti-Branco) and four sowing dates (October, November, December, and January) in 2011 and 2012. The results of the study showed that genotype ANA 5011 had the earliest maturity, while the Moti genotypes had the latest maturity in all sowing dates. The Moti genotypes were found to have greater plant height and 1,000-grain weight than the other two genotypes. In contrast, the Moti genotypes had fewer panicles m^−2^, fewer total filled and total numbers of spikelets panicle^−1^, and lower final yield. The genotype AN-Cambará had the highest number of tillers, filled and total number of spikelets panicle^−1^, panicle m^−2^, and the highest yield. Sowing in either November or December was found to be the most suitable dates for rice cultivation for all genotypes. In conclusion, the AN-Cambará genotype was found to have the highest yield potential for the region among all genotypes studied.

## Introduction

Rice (*Oryza sativa* L.) is a significant source of calories, protein, and nutrients in the Brazilian diet^1^. However, the recent fluctuation in rice production has limited the country’s ability to produce its own demand and importation of this cereal from overseas has become a requirement to meet the domestic demand^2^. Strategies aimed at improving management and production have received great level of attention in recent years and as much as 50% of the total rice production has now been observed in upland production systems (up to 2,000 million hectares under production in any given year)^3–5^. The strategies most studied include flooded and upland production systems, different population stands, genotypes with varying length of growing season, and different sowing dates. Most of these strategies are effective; however, the cost of production under some situations can present a problem for sustainable rice production^6^. Therefore, the development of sustainable and affordable cropping systems that offer maximum yield at a low production cost is needed for wide adoption and increased productivity. Increased rice production in Brazil could lead to a decrease in the amount of rice being currently imported and help boost the economy.

Unlike the environmental factors of air temperature^7^, moisture^8^, weeds, insects, pathogen spectrum, quantity of sunlight, and precipitation, sowing date is one factor correlated to rice grain yield that is easiest for producers to manipulate^9,10^. This is because the culture is highly dependent upon environmental conditions for their appropriate development^11^. In Brazil, rice crop fields located in areas that receive adequate rainfall (between 800 to 1200 mm yr^−1^) between the months of September to April can provide the potential for double cropping. Early rice sowing with a cultivar of early harvest (90–100 days) favors the use of a second crop in same production area. Successive sowings can help maintaining sustainability in upland rice production; for example, unused fertilizer by the first crop can be utilized for the second crop during the same agricultural season^12,13^.

The maximum yield potential of a rice crop is usually achieved when the crop is exposed to the most appropriate temperature range, which can be controlled by sowing at the proper time^2,3^. The decreasing trend in grain yield with delayed sowing date might be associated with the reported significant lower number of filled grains per panicle, lower number of panicles m^−2^, and lower 1,000-grain weight^14^. To improve the yield potential of rice production in certain regions of Brazil, the optimum sowing time and best genotype for each sowing time still needs to be determined^15,16^. For example, in Sri Lanka, four rice crops can be achieved in one year. The rice genotypes Bg 300 and Bg 359 can be cultivated for the first crop during the first inter-monsoon from March-April, the second crop during the south-west monsoon from May to August, the third crop during the second inter-monsoon from September-October, and the fourth crop during the north-east monsoon from November-February^17^. In Faisalabad, Pakistan, the rice genotype “Super Basmati” was seeded during the first and third week of June and first week of July for two years (2008 and 2009)^16^. The authors reported that sowing in the first week of June and harvesting in the first week of November resulted in greater 1,000 kernel weight and also the highest yield^16^. At the Louisiana State University Agricultural Center’s Rice Research Station, Blanche and Linscombe^18^ tested eight rice cultivars planted at seven different sowing dates between the years 2003 to 2005. The authors reported that in the field trials the optimum date for planting rice was April 15, and the genotypes Cypress and CL161 were among the most stable cultivars exhibiting the highest grain and whole kernel milling yields.

Rice plants show better development under temperatures ranging from 22 to 30 °C throughout the growing season. In addition, rice is sensitive to water stress especially between 20 days before and 10 days after flowering. Water stress during the growing season can reduce plant height, tillering, and leaf area, factors that are directly correlated with final yield^19^. Genotype choice is also crucial to a rice crop success as nutritional demand, plant stand, and sowing date changes based on the different genotypes. The selection of genotypes with greater adaptation to different production systems is considered one of the most appropriate practices to achieve adequate yield^20^. The upland rice cultivation is an alternative to meet the Brazilian consumer market needs and potentially generate surpluses that could be exported. The genotypes with low to intermediate height, short and straight leaves, short maturity, high yield potential and good cooking behavior traits are desired for upland rice production^19^. Therefore, the objectives of this study were to determine the best sowing date and yielding genotypes, among four genotypes, for upland rice production in the Ribeira Valley region in São Paulo State (Brazil).

## Results and Discussion

Rainfall and temperature recorded during the time when the trial was conducted was within the long-term average, in general (Table 1). With the exception for a wetter year than normal in the 2011/2012 season (Table 1). The higher than normal precipitation totals in the 2011/2012 season was due to higher than normal rainfall events in October 2011, May and June 2012 (Table 1). All genotypes had similar emergence rates with 50% of plants emerging between 8 and 9 days after planting, which is within the average emergence for dry land rice (Gealy *et al*.^21^) (Table 2). Days needed for flowering and harvest, however, depended on the maturity of each genotype and was shorter for the ANA 5011 and longer for the Moti Amarelo and Moti Branco regardless of sowing date and year (Table 2). ANA 5011 took on average 68 days to reach flowering and 101 days for harvest; while the Moti Amarelo took up to 88 days to reach flowering and 126 days for harvest regardless of sowing date and year (Table 2).

**Table 1:** Mean monthly rainfall and air temperature for the 2011/12 and 2012/13 growing seasons and the 35-year average at Registro, São Paulo State, Brazil.

Month	Rainfall (mm)			Temperature (°C)		
	2011/12	2012/13	35-year	2011/12	2012/13	35-year
			Average^†^			Average
September	05	42	32	18.2	20.3	19.4
October	225	108	120	21.6	22.4	21.5
November	102	97	115	20.9	22.1	21.3
December	234	198	184	23.3	26.6	24.4
January	277	224	218	23.6	24.5	24.6
February	185	132	151	26.4	25.8	25.7
March	71	153	98	23.4	24.2	24.1
April	93	47	77	22.5	22.7	22.3
May	121	71	65	19.7	21.2	20.8
June	240	138	129	18.1	20.1	19.3
Total	1553	1210	1189	—	—	—

^†^Average for the 35-year period 1978–2013.

**Table 2:** . Days needed for emergence, flowering, and harvest in upland rice genotypes under different sowing dates. Registro, São Paulo State, Brazil.

Genotype	Emergence		Flowering		Harvest	
	2011/12^†^	2012/13	2011/12	2012/13	2011/12	2012/13
	October					
ANA 5011	9^¶^	8	70^†^	71	102^§^	100
AN Cambará	9	8	78	79	119	115
Moti Amarelo	9	8	96	97	131	134
Moti Branco	9	8	98	99	131	136
	November					
ANA 5011	8	9	69	71	100	105
AN Cambará	8	9	76	78	113	112
Moti Amarelo	8	9	89	89	125	122
Moti Branco	8	9	90	91	125	125
	December					
ANA 5011	8	9	65	67	98	96
AN Cambará	8	9	68	74	109	110
Moti Amarelo	8	9	81	84	121	116
Moti Branco	8	9	82	84	121	116
	January					
ANA 5011	8	8	65	68	104	102
AN Cambará	8	8	78	78	112	111
Moti Amarelo	8	8	80	85	122	125
Moti Branco	8	8	80	83	127	125

^¶^Standard error is 1 day.

^†^Standard error is 3 days.

^§^Standard error is 4 days.

The majority of the parameters measured in this study during the two cropping seasons were significantly (*P* < 0.10) affected by the 3-way interaction of year by sowing date by genotype tested (Table 3). In the cases where the 3-way interaction was not significant, one or all of the 2-way interactions were significant (Table 3). Therefore, the results presented in this section focus on the interactions that were significant for each parameter.

**Table 3:** Summary of statistical analysis using repeated measures with *Year* as the *repeated* variable for all variables measured in this study. Main effects or their interactions were assumed significant when *P* < 0.10.

Effect	Height	Diameter	Tiller	Lodging	Full Spikelet	Empty Spikelet	Panicle m^−2^	1,000 Seed Weight	Yield
Year	0.0007	0.0001	0.0001	0.0001	0.0001	0.0001	0.0001	0.0011	0.0001
Sowing	0.0001	0.0001	0.0001	0.0001	0.0001	0.0001	0.0001	0.0116	0.0001
Genotype	0.0001	0.0004	0.0109	0.0001	0.0001	0.0001	0.0001	0.0001	0.0001
Year * Sowing	0.0001	0.0001	0.0001	0.0001	0.0265	0.0001	0.0001	0.0013	0.4009
Year * Genotype	0.0027	0.4577	0.3734	0.0001	0.0116	0.2474	0.007	0.4602	0.0219
Sowing * Genotype	0.0006	0.5601	0.1244	0.0001	0.656	0.0043	0.0001	0.0034	0.0029
Year * Sowing * Genotype	0.0693	0.0116	0.0031	0.0001	0.4804	0.0289	0.0001	0.0001	0.0466

Although there was a significant 3-way interaction for plant height and diameter, these two properties were found to be only slightly correlated to the productivity parameter measured including final yield (r = 0.59, p-value = 0.01; and r = 0.46, p-value = 0.01, for height and diameter, respectively), number of tillers (r = 0.54, p-value = 0.01; and r = 0.57, p-value = 0.01;, for height and diameter, respectively), and number of panicles m^−2^ (r = 0.51, p-value = 0.01 and r = 0.43, p-value = 0.01, for height and diameter, respectively). Plant height was closely related to maturity with short season genotypes resulting in the shorter plants. The ANA 5011 averaged 93 cm and 82 cm, the AN Cambará averaged 94 cm and 97 cm, the Moti Amarelo averaged 114 cm and 110 cm, and the Moti Branco averaged 112 cm and 106 cm in the 2011/2012 and 2012/2013, respectively. In general, the sowing that took place in January led to the shortest plants (average 95 cm), while all three other sowing dates had higher heights (average 103 cm). Rodenburg *et al*.^22^ reported that sowing date caused a reduction in as much as 11 cm among the varieties tested in Guinea Savana. Similar to plant heights, plant diameters were thinnest in the last sowing (5.3 mm) compared with the other three sowing dates (6.7 mm). In this study, there were also poor correlations between number of tillers and final yield (r = 0.39, p-value = 0.05), 1,000 seed weight (r = −0.05), and number of panicles m^−2^ (r = 0.32). As observed for the diameter, the number of tillers was highest in the first two sowing dates (average 2.3 tillers per plant) and lowest in the last two sowing dates (1.4). In addition, plant height, diameter, and number of tillers were also poorly correlated among each other (r values of 0.32, 0.53, and 0.56, for height and diameter, height and tiller, and diameter and tiller, respectively), suggesting that those parameters are a poor estimator of rice production potential under conditions similar to those observed during this study.

Lodging was an issue primarily for the Moti genotypes when planted in November (92% for Moti Amarelo and 69% for Moti Branco) in the first season or planted in November (92% for Moti Amarelo and 78% for Moti Branco) and December (87% for Moti Amarelo and 77% for Moti Branco) in the second season. The lack of a clear trend between lodging and plant height and diameter suggests that lodging is more closely related to the genotypes than to the growing conditions observed during this study. Islam *et al*.^23^ reported that dry weight per unit length and breaking resistance of the lower internodes are the main components associated with lodging.

Panicles m^−2^ varied considerably depending on the genotype, sowing date, and growing season (Table 4). In general, the highest number of panicle m^−2^ was observed for the sowing in November and December in both years. Sowing in January was found to have the lowest number of panicles m^−2^ in 2012; in contrast, sowing in October was found to have the lowest number of panicles m^−2^ in 2012/2013 (Table 4). Many environmental factors could be at play determining the number of panicles per m^−2^ for rice production. For example, Sartori *et al*.^6^, investigated the effects of different sowing dates in southern Brazil during the crop season 2011/12. The authors observed a reduction in the number of panicles m^−2^ as a result of delaying sowing date to December compared to the region’s recommended sowing date of September, which was attributed to temperature changes between September and December. In this study, the highest number of panicles m^−2^ tended to be observed for the genotype ANA 5011 when sowing in November or December (Table 4). The AN Cambará tended to have the second highest number of panicles m^−2^, and both Moti genotypes had, in general, the lowest number of panicles m^−2^ (Table 4). Other researchers have reported that the number of panicle m^−2^ not always relates to yield and cultivars with high number of panicles m^−2^ have yield that is lower than cultivars with lower number of panicle m^−2^ (Nakano and Tsuchiya^19^).

**Table 4:** Mean separation for rice panicle m^−2^ for all genotypes studied as function of year and sowing date.

Genotype	2011							
	October		November		December		January	
	panicles m^−2^							
ANA 5011	201^†^	jklmn	229	def	265	bc	177	op
AN Cambará	209	ghijkl	235	de	248	cd	174	p
Moti Amarelo	184	mnop	217	efghij	203	ijklm	143	q
Moti Branco	181	nop	220	efghij	197	klmn	142	q
	2012							
	**panicles m** ^**−2**^							
ANA 5011	175	p	286	a	228	efg	197	klmno
AN Cambará	191	lmnop	281	ab	275	ab	220	efghi
Moti Amarelo	196	lmno	210	fghijkl	218	efghij	225	efghi
Moti Branco	202	ijklmn	216	efghijk	232	de	208	hijkl

^¶^Any mean followed by the same lowercase letters are not significantly different (*P value* > 0.10).

^†^Standard error is 7 panicles m^−2^.

In general, sowing in January significantly reduced the number of full spikelets per panicle (Table 5), while sowing at any other time led to similar number of full spikelets (Table 5). The only exception was sowing in December in the second growing season, which had a small but significant greater number of spikelets compared with the October and November sowing (Table 5). In general, the genotype AN Cambará (average 89 and 110 full spikelets in 2011/12 and 2012/13, respectively) had the highest number of full spikelets per panicle and the Moti types (average between the two varieties of 73 and 93 full spikelets in 2011/12 and 2012/13, respectively) had the lowest in both cropping seasons (Table 5). Iqbal *et al*.^24^ in 2004 and 2005, tested the effects of transplanting date for two varieties (Super Basmati and Basmati 2000) (1^st^ week of July and 3^nd^ week of July) in three different regions of Pakistan (Faisalabad, Kalashah Kaku and Gujranwala). The authors reported higher number of number of full spikelets per panicle when the variety was Basmati 2000, they also reported higher 1,000 grain weight for this variety. Early planting favored the number of full spikelets per panicle and the rice grain yield.

**Table 5:** Mean separation for number of full spikelets per panicles as a function of sowing date by year and genotype by year.

Sowing Date	2011		2012	
	full spikelet			
October	84^†^	c	100	b
November	83	c	101	b
December	82	c	108	a
January	67	d	85	c
Genotype	2011		2012	
	**full spikelet**			
ANA 5011	80	d	98	b
AN Cambará	89	c	110	a
Moti Amarelo	72	e	98	b
Moti Branco	73	e	87	c

^¶^Any mean for Sowing date or Genotype followed by the same lowercase letters are not significantly different (*P value* > 0.10).

^†^Standard error is 2 full spikelet.

For the first cropping season, rice 1,000 grain weight was highest for the Moti genotypes when sowed in November (32.9 g and 36.1 g for Moti Amarelo and Branco, respectively) and January (29.5 g and 32.1 g for Moti Amarelo and Branco, respectively), while sowings in October (average 25.8 g) and December (average 27.2 g) led to similar 1,000 grain weights among all four genotypes (Table 6). In contrast, in the second cropping season, the 1,000 grain weight was greater with the Moti genotypes in the first three sowings (averaging 29.3 g for the Moti genotypes and 24.2 g for the others), while sowing in January led to similar 1,000 grain weights among all genotypes (Table 6). The difference in the rice 1,000 grain weight observed for the Moti genotypes is likely due to the fact that Moti have long, thick grains while the ANA 5011 and AN Cambará have long, thin grains. Sfadar *et al*.^25^ investigated eight medium grain rice accessions and five different sowing dates (April 16^th^, May 1^st^, May 16^th^, June 1^st^ and June 16^th^) between 2004 a 2006. The authors reported that the majority of the accessions had higher 1,000 grain weight when planted on May 16^th^. In addition, rice grain yield and number of full spikelets per panicle increased with the later planting, whereas days to 100% flowering and plant height decreased.

**Table 6:** Mean separation for rice 1,000 grain weight for all genotypes studied as function of year and sowing date.

Genotype	2011							
	October		November		December		January	
	g							
ANA 5011	25.7†	ijklm	25.5	jklm	26.4	ghijlkm	27.6	efghijk
AN Cambará	25.2	jklm	23.9	lm	25.8	ijklm	23.5	m
Moti Amarelo	27.7	efghijk	32.9	b	27.6	efghijk	29.5	cdef
Moti Branco	24.9	klm	36.1	a	28.9	defgh	32.1	bc
	2012							
	**g**							
ANA 5011	24.4	lm	24.4	lm	25.0	jklm	26.2	hijklm
AN Cambará	24.1	lm	23.7	lm	24.0	lm	25.6	ijklm
Moti Amarelo	27.9	defghij	29.2	cdefgh	29.2	cdefgh	25.6	ijklm
Moti Branco	28.5	defghi	30.0	bcd	30.7	bcd	26.6	fghijkl

^¶^Any mean followed by the same lowercase letters are not significantly different (*P value* > 0.10).

^†^Standard error is 1.1 gram.

Rice grain yield was, in most cases, greater in the second (overall average 3,441 kg ha^−1^) cropping year than in the first (overall average 2,442 kg ha^−1^) cropping year for all genotypes (Table 7). Sowing in the months of November (average of 3,118 in 2011/12 and 4,299 kg ha^−1^ in 2012/13) and December (average of 2,947 in 2011/12 and 3,634 kg ha^−1^ in 2012/13) resulted in the highest rice yield potential compared with sowing in October (average of 2,148 in 2011/12 and 3,228 kg ha^−1^ in 2012/13) or January (average of 1,557 in 2011/12 and 2,610 kg ha^−1^ in 2012/13) (Table 7). In the first cropping season, the genotype AN Cambará consistently had the highest yield averaging 3,373 kg ha^−1^, while the Moti Branco, in most cases, had the lowest yield averaging 1,799 kg ha^−1^ (Table 7). In the second cropping season, the genotypes ANA 5011 and AN Cambará were the highest yielding, though not always significantly greater than the Moti genotypes (Table 7). Akbar *et al*.^26^ found that sowing date had a significant effect on rice yield in Pakistan. The authors reported that increases in yield were likely due to favorable weather conditions (such as accumulated rainfall and temperature) during the critical stages of crop development. Others (Lack *et al*.^27^) have reported that optimum rice yield potential can only be achieved when the crop vegetative and reproductive stages match ideal environmental conditions such as temperature, solar radiation, and rainfall. Lack *et al*.^26^ investigated the effects of sowing date on three rice cultivars in Khouzestan (South-west Iran) in the 2010. The authors reported that the highest grain yield was obtained for Danial and Hamar varieties with averages of 5,591 and 5,549 kg ha^−1^. They also reported that sowing date had a significant effect on grain yield, 1000 grain weight, the number of filled grains per panicle and the number of fertile tillers; rice was planted on May 5^th^, May 25^th^, and June 15^th^. Planting on May 25^th^ was found to result in the highest grain yield with an average of 6018.3 kg ha^−1^ ^27^. Slaton *et al*.^28^ suggested that modern rice cultivars produced maximum grain yield when seeded from February 16^th^ through March 28^th^ at Crowley, LA, and through March 29 and April 26 at Stuttgart, AR. In a different study, Vange and Obi^29^ studied several rice cultivars in Benue, Nigeria, during two growing seasons and reported that planting on June 15^th^ resulted in the highest yield, 2,700 kg ha^−1^, at Makurdi and June 30^th^, 3320 kg ha^−1^, at Otobi. The authors also reported that delaying planting by 12 to 15 days could result in a 5% decrease in yield.

**Table 7:** Mean separation for rice final yield for all genotypes studied as function of year and sowing date.

Genotype	2011							
	October		November		December		January	
	kg ha^−1^							
ANA 5011	1824	lmn†	2461	ijkl	3833	bcde	1545	mno
AN Cambará	3759	cde	3551	cdefg	3693	cdef	2488	ijkl
Moti Amarelo	1812	lmn	3187	defghi	2381	jkl	1351	no
Moti Branco	1196	no	3272	defgh	1881	lmn	845	o
	2012							
	**kg ha** ^**−1**^							
ANA 5011	3566	cdefg	4602	ab	4087	abc	2546	hijkl
AN Cambará	3520	cdefg	4705	a	3877	bcd	3086	efghij
Moti Amarelo	2945	fghijk	4096	abc	3472	cdefg	2256	klm
Moti Branco	2879	ghijk	3795	cde	3100	efghij	2552	hijkl

^¶^Any mean followed by the same lowercase letters are not significantly different (*P value* > 0.10).

^†^Standard error is 275 kg ha^−1^.

### Implications of this work’s findings

The results of this research showed that the ANA 5011 had the earliest maturity among the all genotypes studied, with average days to reach flowering of 68 d and to reach harvest of 101 d. In contrast, the Moti genotype was found to have the latest maturity among all genotypes studied needing as much as 88 d to reach flowering and as long as 126 d to reach harvest. Also, the Moti genotypes had the tallest plants and 1,000-grain weight (average 29 g) but lower panicles m^−2^ (average 199 panicles m^−2^), lower full spikelets (average 82 full spikelets per panicle), lowest total spikelets panicle^−1^, and lower average yield (average 2,564 kg ha^−1^) compared with the other two (average 3,321 kg ha^−1^) genotypes. The AN Cambará had the highest number of tillers, full spikelets (average 100 full spikelets per panicle), and panicle m^−2^ (average 229 panicles m^−2^), and the highest rice grain yield (average 3,585 kg ha^−1^), suggesting that this genotype would be the best option for rice cultivation in the Ribeira Valley, São Paulo State, Brazil.

Sowing in January likely limited plant vegetative and reproductive development as indicated primarily by the lowest yield observed (average 2,083 kg ha^−1^). In contrast, sowing in November (yield average 3,709 kg ha^−1^) and December (yield average 3,291 kg ha^−1^) was found to be the most suitable for rice cultivation in the Ribeira Valley, São Paulo State, Brazil.

## Material and Methods

The experiments were performed in Registro city, São Paulo state, Brazil, at geographical coordinates of 24° 31’ South and 47° 51’ North and altitude of 25 m above sea level. The slope in the experimental area is between 0 and 12%, the climate type according to Koeppen classification is humid subtropical with hot summers (Cfa). The average annual temperature is 27 °C with an annual rainfall of 1,500 mm. The soils are described as alluvial and clayey soils of Eutrophic Cambisols type^30^ that correspond to Eutrophic Inceptisol^31^. Soil samples were collected in June 2011 for characterization of chemical properties from the depth of 0 to 15 cm with four replicates being collected (Table 8).

**Table 8:** Summary of soil chemical properties at the depth of 0 to 15 cm. Registro-SP, 2011.

P	OM (%)	pH (CaCl_2_)	K	Ca	Mg	H + Al	Al	CTC
ppm			mmol_c_ kg					
5 ± 0.5	2.8 ± 0.4	4.2 ± 0.2	0.84 ± 0.2	14.4 ± 0.8	4.8 ± 0.6	76.8 ± 1.3	15.6 ± 0.6	97.2 ± 2.7

This study was set up in a completely randomized block design with four replications for two consecutive growing seasons (2011/12 and 2012/13) in the same experimental area. Four upland rice genotypes (ANA 5011-short season, AN Cambará-mid season, Moti Amarelo and Moti Branco-both late seasons genotypes) and four sowing dates were tested in a full factorial design having a total of 64 experimental plots. In the first growing season, planting took place on October 22, 2011, November 19, 2011, December 16, 2011, and January 14, 2012, respectively. In the second growing season, planting took place on October 22, 2012, November 19, 2012, December 20, 2012, and January 14, 2013, respectively. In both growing seasons, the planting rate was 75 kg of seeds ha^−1^. Plant emergence was collected between 8 and 9 days after sowing in both growing seasons (Table 1). Each experimental plot had five 7 m long planting rows, spaced at 0.35 m intervals. However, the plot area used for sampling, data collection, and grain harvest was comprised of the center 6 m and only for the three central rows. Prior to planting, tillage operations included moldboard plow to a depth of 0.3 m followed by a disc harrow to prepare the seedbed in August 2011 and August 2012. Based on the soil analysis, limestone (2.6 t ha^−1^) was applied (August 2011) to reach 50% base saturation and was incorporated using the disc harrow^32^. In each plot, nutrient application was performed manually by adding 600 kg ha^−1^ of the blended fertilizer 04–14–08 (N-P-K). Top-dress fertilizer application was conducted at three different times during each growing season. In both years, the first top-dress application was performed at 18 days after emergence (DAE) applying 25 kg ha^−1^ N as ammonium sulfate (21% N and 24% S); the second at 35 (DAE) using 25 kg ha^−1^ N of a blended fertilizer containing 20-00-20 (N, P, and K); and the third at 50 (DAE) with 10 kg ha^−1^ N as urea (46-0-0). In the first growing season, fungicide was applied three times: two with azoxystrobin at 0.4 L commercial product (c.p.) ha^−1^ and one with tebuconazole at 0.15 L ha^−1^ for control of *Pyricularia grisea* and *Bipolaris oryzea*. In the second growing season, two fungicide applications were required: one with azoxystrobin and one with tebuconazole for the same plant diseases at the same rates as above.

When the grains had reached physiological maturity, evaluation of crop development was performed in *situ* by randomly examining 10 plants within each plot. The measurements collected included plant height (cm) measured from the soil surface to the panicle tip; stem diameter (mm) measured using a caliper at 1 cm height from the soil surface; number of tillers per plant determined by direct count; lodging degree (%) determined by visual observations using a scoring range (0, no lodging; 1, up to 5% lodged plants; 2, 5 to 25%; 3, 25 to 50%; 4, 50 to 75%; and 5, 75 to 100%^33^). The number of panicles m^−2^ was obtained by counting the number of panicles present within a linear 2 m in two central rows from each plot. Full, empty, and total number of spikelets panicle^−1^ was obtained by counting the number of spikelets in 20 panicles collected at harvest from each plot. The 1,000-grain weight (g) was assessed by random collecting and weighing of four 1,000-grain subsamples from each plot. Grain yield (kg ha^−1^) was determined by weighing husked grains from the entire useful plot area (three central rows 6 m long). In this study, the reported grain yield was corrected to moisture content of 13%.

The main effects of genotype, planting date, growing season, and their interaction were assessed using repeated measure (growing season as the repeated variable) analysis using Proc Mixed in SAS 9.3 (Littell *et al*.^34^). The Akaike information criteria (AIC) value was used as the model selection criteria to determine the best covariance model for the repeated variable. Significance of differences among the variables measured (*P* ≤ 0.10) were determined by mean separation using Fisher’s least significance difference test (LSD). We chose the probability level *P* ≤ 0.10 so that we would allow for errors related to field variability to not interfere in the analysis. All data analyses were performed on replicate data, and the results are presented as the average of four replicates. The data set was analyzed for the presence of outliers before any statistical test was conducted and no outliers were detected.
